# Clinical utility of nuclear imaging in the evaluation of pediatric adrenal neoplasms

**DOI:** 10.3389/fonc.2022.1081783

**Published:** 2023-01-17

**Authors:** Christelle Fargette, Barry Shulkin, Abhishek Jha, Karel Pacak, David Taïeb

**Affiliations:** ^1^ Department of Nuclear Medicine, La Timone University Hospital, Centre Européen de Recherche en Imagerie Médicale (CERIMED), Aix-Marseille University, Marseille, France; ^2^ Department of Diagnostic Imaging, St. Jude Children’s Research Hospital, Memphis, TN, United States; ^3^ Section on Medical Neuroendocrinology, Eunice Kennedy Shriver National Institute of Child Health and Human Development, National Institutes of Health, Bethesda, MD, United States

**Keywords:** adrenal, pediatric neoplams, PET, nuclear imaging, neuroblastoma, cushing, pheochromocytoma, pediatric neoplams

## Abstract

Adrenal neoplasms rarely occur in children. They can be diagnosed in the presence of endocrine, metabolic or neurological problems, an abdominal mass, more rarely an adrenal incidentaloma, or in the context of an adrenal mass discovered in the evaluation of childhood cancer including hematologic malignancy. According to standard medical practice, pediatric malignancies are almost always evaluated by ^18^F-fluorodeoxyglucose positron emission tomography with computed tomography ([^18^F]FDG PET/CT). Nuclear imaging using specific radiotracers is also an important tool for diagnosing and staging neuroblastoma, pheochromocytoma, hormone hypersecretion, or indeterminate adrenal masses. The Hippocratic oath “primum non nocere” encourages limitation of radiation in children per the ALARA concept (as low as reasonably achievable) but should not lead to the under-use of nuclear imaging because of the potential risk of inaccurate diagnosis or underestimation of the extent of disease. As in adults, nuclear imaging in children should be performed in conjunction with hormone evaluation and morphological imaging.

## Background

Adrenal gland neoplasms are rarely found in the pediatric population and are usually revealed by abdominal pain or palpable mass. Adrenal lesions include a wide diversity of conditions such as congenital, neoplastic, infectious, and traumatic lesions. Although a definitive diagnosis is obtained by pathological analysis, anatomical imaging techniques such as ultrasonography (US), magnetic resonance imaging (MRI) or computed tomography (CT) play a crucial role in tumor characterization and staging. The age of the patient, clinical history, and laboratory findings can further help to narrow down the differential diagnosis. Nuclear medicine studies are indicated in select clinical scenarios regardless of secretion, hormonally active lesions or indeterminate large adrenal masses. Recognition of the imaging features of these lesions are important because it can guide the treatment approach and may eliminate unnecessary invasive procedures.

## Neuroblastomas

Neuroblastomas (NBs) are the most common extracranial solid malignancy of childhood (1, 2), comprising approximately 85% of pediatric adrenal malignancies. They are derived from neural crest progenitor cells and can occur anywhere along the sympathetic nervous system (3). The median age at presentation is 17 months (3, 4), and up to 50% of cases occur in the first months of life. Clinically, neuroblastoma patients who have localized disease are often asymptomatic but may be detected incidentally on imaging (1). It can present as a palpable mass and can cause abdominal distension or pain (1). When symptomatic, children typically present with signs related to direct tumor growth or invasion into the neighboring structures or with symptoms secondary to metastatic disease (i.e., intradural or epidural extension and may cause neurologic impairments or skeletal pain due to bone metastases) (1). In addition, symptoms related to catecholamines, to vasoactive inhibitory and other peptides overproduction, or to cerebellar paraneoplastic syndrome can rarely be observed (1).

**Figure 1:**
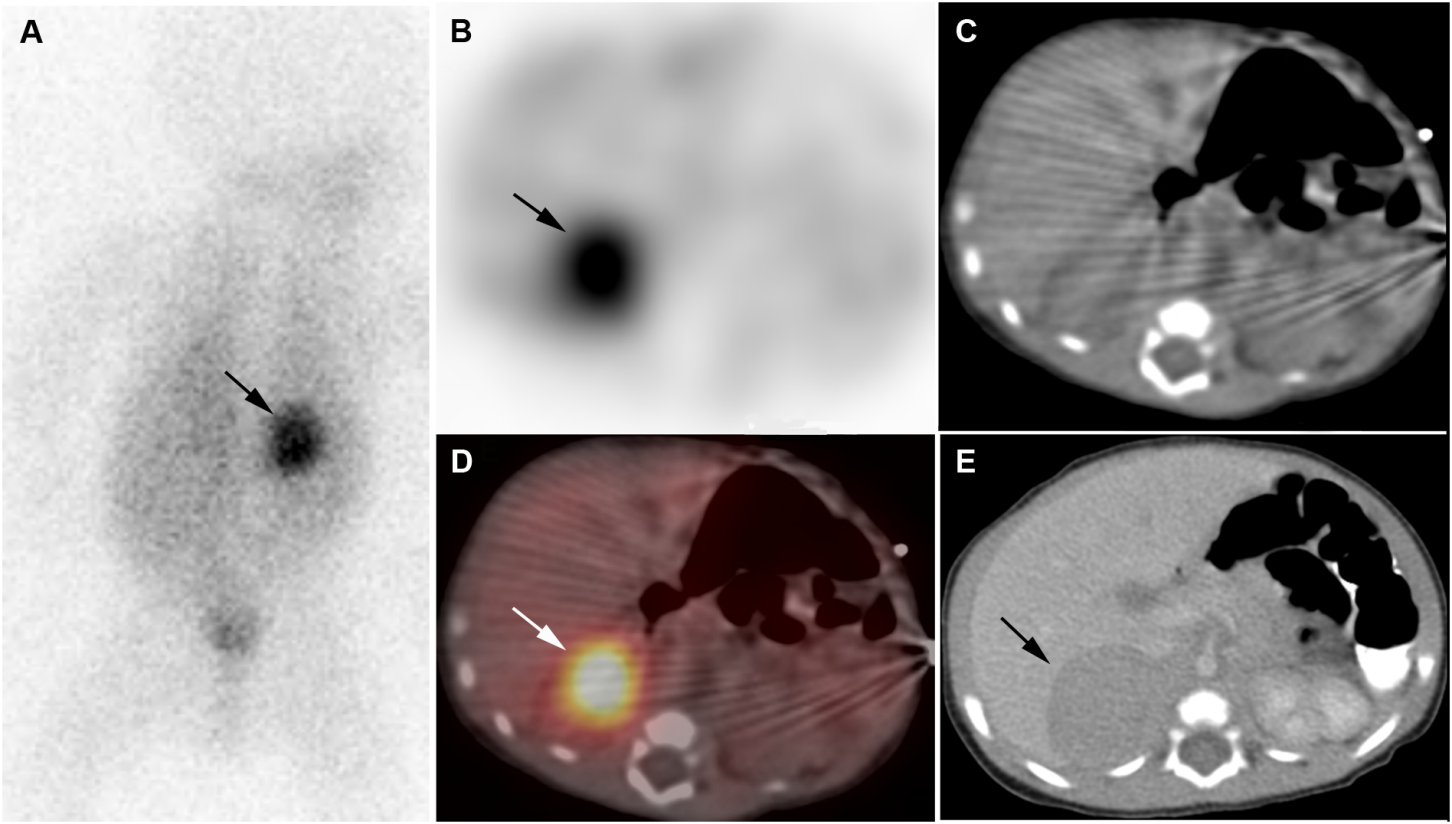
Neuroblastoma. 2-week-old boy with neuroblastoma. [^123^I]I-mIBG posterior planar image **(A)**, [^123^I]I-mIBG SPECT transverse image **(B)**, CT for attenuation correction **(C)**, [^123^I]I-mIBG SPECT/CT fusion image **(D)**, Diagnostic CT **(E)**. Intense uptake in right adrenal mass on [^123^I]I-mIBG scan (arrows).

Imaging allows assessment of disease extension and resectability. The initial suspicion for neuroblastoma arising from the adrenal gland or retroperitoneum usually follows US or cross-sectional imaging. MRI may be preferable, in principle, because it is free of ionizing radiation and superior in evaluation of intraspinal and marrow involvement; however, CT scan is more widely available and is a rapid technique enabling sedation avoidance. Because NB cells express the cell membrane norepinephrine transporter, patients with NB can be evaluated by using [^123^I]I-metaiodobenzylguanidine ([^123^I]I-mIBG), which is recommended as the first-choice radiopharmaceutical for diagnosis, staging, and restaging (5, 6) (**Figure 1**). Skeletal extension can be staged on [^123^I]I-mIBG according to the SIOPEN or Curie scoring systems. L-3,4-Dihydroxy-6-[^18^F]fluorophenylalanine (6-[^18^F]FDOPA) can surpass [^123^I]I-mIBG (7–11), but the latter is still required before inclusion in therapeutic clinical trials. 6-[^18^F]FDOPA was found to perform better than CT/MRI for assessing bone/bone marrow and nodal lesions but can be inferior to CT/MRI for assessing liver metastases. A study included 21 children with advanced neuroblastoma, mostly studied at restaging, who underwent 6-[^18^F]FDOPA PET/CT (12) and CT/MRI scans (37 paired studies). The findings were concordant in 30 of 37 cases. CT/MRI showed false-negative results in 2 patients with bone lesions that were positive on 6-[^18^F]FDOPA PET/CT. Five false-positive CT/MRI results were attributed to residual masses following surgery or end of treatment or to lymph node enlargement not due to tumors. On a lesion-based analysis, sensitivity of 6-[^18^F]FDOPA PET/CT was higher than that of CT/MRI, 91% and 48%, respectively. The greatest differences in lesion detection were within bone/bone marrow, where 6-[^18^F]FDOPA PET/CT detected 95% of lesions and CT/MRI detected 7%. Additionally, 6-[^18^F]FDOPA PET/CT detected more nodal sites (94%) than did CT/MRI (72%). Liver metastases were more accurately detected by CT/MRI than by 6-[^18^F]FDOPA PET/CT, 100% and 63%, respectively. 6-[^18^F]FDOPA PET/CT uptake in brain metastases has also been described, and the method may detect bone and lymph node metastases that are negative on diagnostic [^123^I]I-mIBG scintigraphy but confirmed on post-treatment [^131^I]I-mIBG imaging. In another study, 55 patients with neuroblastoma underwent 6-[^18^F]FDOPA PET (202 studies), [^18^F]FDG PET (205 studies), and [^123^I]I-mIBG (80 scans) (13). 6-[^18^F]FDOPA PET identified 41 of 42 tumors with viable tumor cells. One false-negative result was identified: the patient had received induction chemotherapy and their histology results showed only scattered tumor cells. A false-positive result occurred in a patient following chemotherapy. Sixteen patients had [^123^I]I-mIBG imaging. Three of four tumors with negative [^123^I]I-mIBG imaging were positive on both 6-[^18^F]FDOPA PET and [^18^F]FDG PET images, whereas one false negative was visualized only on 6-[^18^F]FDOPA PET images. The sensitivity of 6-[^18^F]FDOPA PET imaging was higher than that of [^123^I]I-mIBG imaging (100% vs. 75%, respectively). In 46 tumors, the sensitivity of [^18^F]FDG PET was 87% and specificity was 63%. Four of five false-negative tumors on [^18^F]FDG PET images were positive on 6-[^18^F]FDOPA PET images.

18-Fluorine–labeled analogs of benzylguanidine hold great promise for imaging neuroblastoma (14). [^18^F]FDG can be useful in characterizing [^123^I]I-mIBG -negative or weakly positive tumors characterization and prognostic assessment but can show nontumor uptake. PET agents that target somatostatin receptors (SSTR) have also shown some interesting results and may represent companion agents to determine whether a patient is likely to benefit from peptide receptor radionuclide therapy. Overall, [^123^I]I-mIBG SPECT/CT or 6-[^18^F]FDOPA PET/CT should be performed as first-line imaging investigations, depending on the therapeutic protocol’s requirements.

## Pheochromocytomas

Pediatric pheochromocytomas (PHEOs) occur in children and adolescents having a mean age at diagnosis of approximately 11 years (15). The most common presenting symptoms are headache, diaphoresis, or palpitations (15). Sustained hypertension is seen more often in about 93% of pediatric cases whereas paroxysmal hypertension is observed only in 7% of cases (15). Pediatric pheochromocytomas are most frequently hereditary (up to 80%) and multifocal as compared to their adult-onset PHEOs (15). The underlying genetic background typically contains germline pathogenic variants of the *VHL (*27-32*%), SDHB (*39-44*%), SDHD* (10-16%), *RET* (4%) or *NF1* (1%) genes (15). Other genes are mutated in less than 1% of cases. Approximately 50% of pediatric pheochromocytoma and/or paraganglioma (PGL, together PPGL) patients have a malignant tumor, more specifically in the presence of *SDHB* mutations (15). Recurrences can be observed in up to 30% in the pediatric population (15). In rare cases, PPGL can occur in children in the setting of *NF1*; Carney triad (young females with no familial trait in whom 2 to 3 of the classical tumors [i.e., gastric GIST, pulmonary chondroma, PGL] develop); Carney-Stratakis syndrome (*SDHx*-related PPGL including GIST and PGL); or Pacak-Zhuang syndrome (young females with no familial trait in whom polycythemia develops at an early age, multiple PPGL and duodenal somatostatinoma, presence of somatic *HIF2A* mutation in tumors) (15). In patients with VHL, PHEO usually develops and can be bilateral (15) and associated with extra-adrenal PGL that can arise in a synchronous or asynchronous manner. VHL type 2, which is predominantly associated with *VHL* missense mutations, is defined by the occurrence of PPGL, either alone (type 2C) or with hemangioblastomas (type 2A) or with hemangioblastomas and renal cell carcinomas (type 2B). *SDHB*-mutations typically give rise multiple extra-adrenal and/or PHEO (15). In the setting of *SDHD* mutations, retroperitoneal PGLs are rare and often associated with head and neck PGL. Extra-adrenal PGL can be found in the organ of Zuckerkandl, at the level of the inferior mesenteric artery. Multiple endocrine neoplasia (MEN) type 2B is due to Met918Thr *RET* mutation in >95% cases and includes medullary thyroid carcinoma, PHEO, and extra-endocrine features (16). Penetrance for PHEO is lower (about 40-50%) than for medullary thyroid carcinoma (90%) (16). Bilateral PHEOs are frequent (60-70% of cases), occurring in a synchronous or metachronous manner. VHL PHEOs typically arise in younger patients, as low as 2-years of age, than other subtypes do (15).

Information from the family history, assessment of serum calcitonin, and metanephrines secretion profile can be combined to identify the genetic background (17). Metanephrine secretion (plasma and urine) is low in extra-adrenal locations or *VHL*-related PHEO. In the presence of PHEO, an adrenergic phenotype is most likely to be associated with MEN2 or NF1.

There is no reliable histological system predicting the biological behavior of PPGL, and malignancy is —as for adults— defined by the presence of nodal, bone, or visceral metastasis (17). Metastases are more commonly seen in children compared to adults (15). Overall, malignancy risk is increased in *SDHB* compared to other subtypes and increases with tumor size (17).

**Figure 2:**
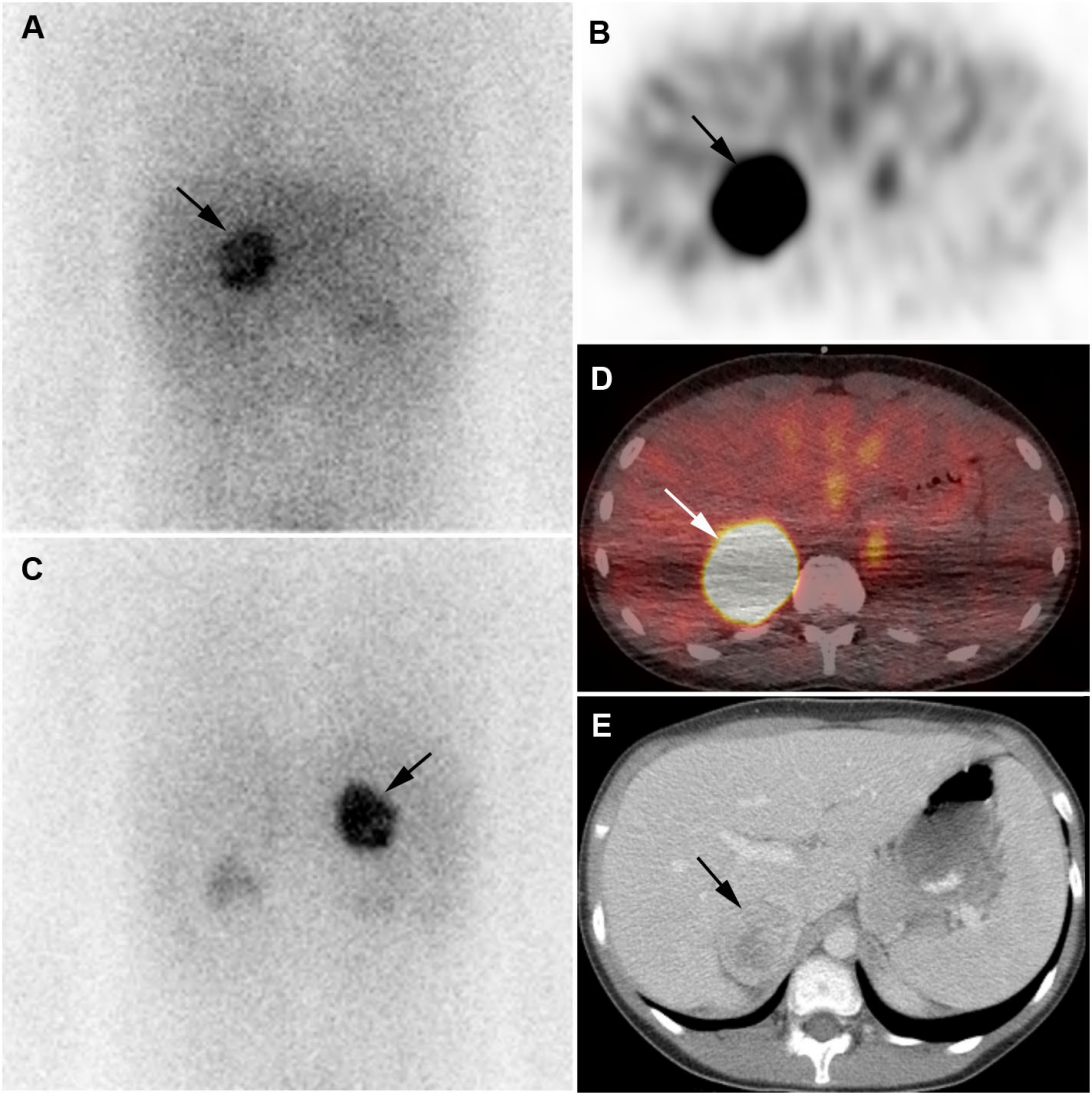
Pheochromocytoma. 11-year-old boy with pheochromocytoma. [^123^I]I-mIBG anterior planar image **(A)**, transverse [^123^I]I-mIBG SPECT **(B)**, [^123^I]I-mIBG posterior planar image **(C)**, Fusion [^123^I]I-mIBG SPECT/CT **(D)**, Diagnostic CT **(E)**. Intense uptake of [^123^I]I-mIBG in right adrenal mass (arrows).

**Figure 3:**
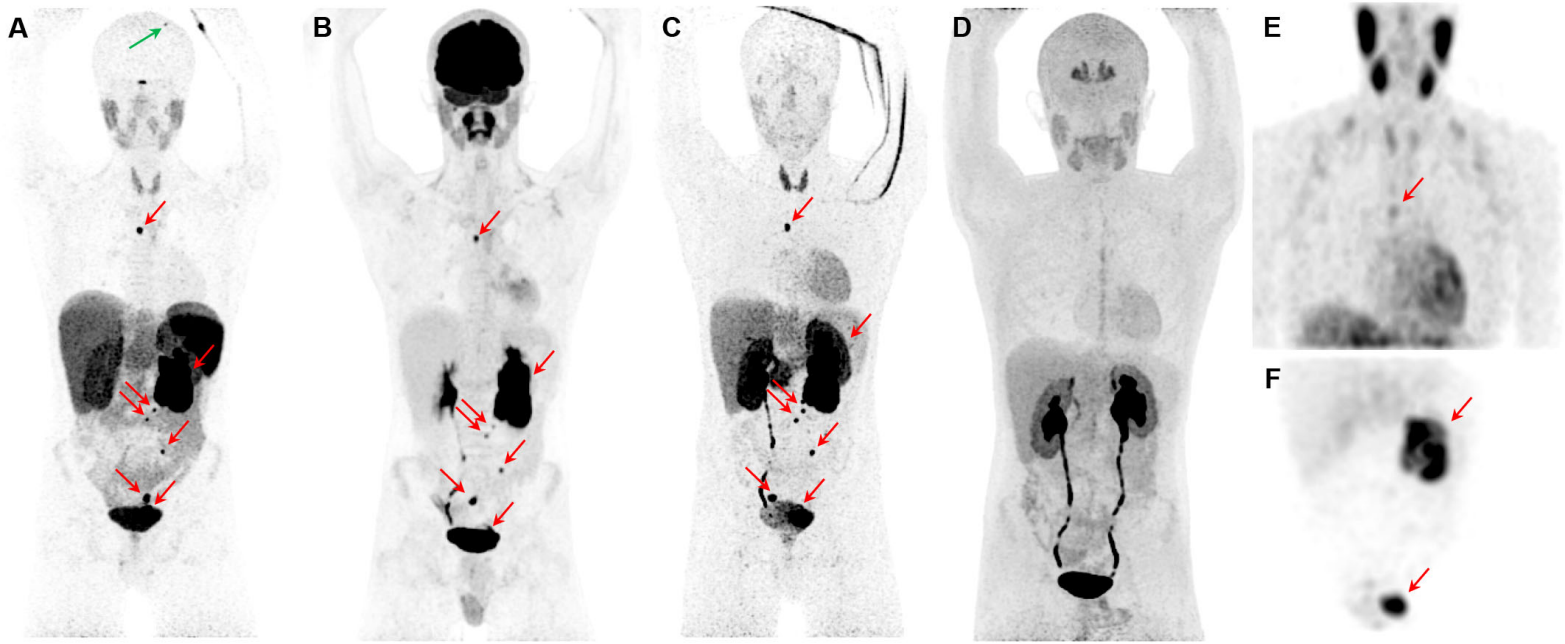
Metastatic pheochromocytoma. The maximum intensity projection images of [^68^Ga]Ga-DOTATATE PET **(A)**, [^18^F]FDG PET **(B)**, ^18^F-fluorodopamine ([^18^F]FDA) PET **(C)**, 6-[^18^F]FDOPA PET **(D)**, and [^123^I]I-mIBG SPECT/CT **(E, F)** of a 16-year-old boy with metastatic PPGL carrying a germline mutation in *SDHB* gene demonstrates a 5.7-cm left lesion in the adrenal/periadrenal region abutting the left upper renal pole along with lesions in the abdomen, mesentery, left peri-rectal and precarinal (red arrows) regions, and skull (green arrow). The [^68^Ga]Ga-DOTATATE PET detects an additional lesion in skull bone (blue arrow); however, 6-[^18^F]FDOPA failed to detect any lesions.

The aim of imaging is to determine whether the disease is resectable, unifocal, or multifocal and to identify metastatic recurrences and association with non-PPGL tumors (18–20). PHEOs typically exhibit an attenuation density on unenhanced CT >10 HU (often > 20 HU) (21, 22). They frequently contain a central area of necrosis and may have a “ring sign” (23), which is peripheral contrast enhancement at the edge of the lesion (observed in 40% of cases compared to 2-3% for other tumors). On contrast-enhanced CT or MR images, PPGLs have increased vascularity and strong enhancement, which can be homogeneous in small tumors or heterogeneous in larger ones due to cystic, necrotic, and/or hemorrhagic components (22). They can exhibit a classical but inconsistently increased signal intensity on T2-weighted images (22, 24). Due to their rarity, a retroperitoneal mass in children can be suspicious for Wilms tumor or neuroblastoma rather than for PPGL. Nuclear imaging often has limited clinical impact in MEN2 and NF1 despite high sensitivities and specificities. By contrast, it complements morphological imaging in *VHL-* and *SDHx*-patients for multifocality screening and detection of metastases. Head-to-head comparative studies of radiopharmaceuticals are lacking in children, but it is widely accepted that imaging phenotype is driven mostly by tumor location and genetic background. Thus far, two functional imaging studies have been performed in pediatric PPGL patients (25, 26). In the first study, SSTR PET/CT, with a detection rate of 93.5% performed significantly better than [^18^F]FDG (79.4%) and CT/MRI (73.8%) in 9 metastatic *SDHx*-related PPGL patients (25). In the second study in 32 pediatric PPGL patients, SSTR PET/CT as well as CT had a detection rate of 100% and performed better than [^123^I]I-mIBG (82.4%), [^18^F]FDG (66.7%) in detecting primary tumors (26). However, in the overall detection rate of both primary and metastatic PPGL, SSTR PET/CT (95.2%) performed better than [^123^I]I-mIBG (65.0%), [^18^F]FDG (80.0%), and CT (91.4%) did (26). However, in both studies, two soft-tissue metastatic abdominal lesions were missed by SSTR PET/CT but detected by [^18^F]FDG. Further, based on adult experience, 6-[^18^F]FDOPA (or [^123^I]I-mIBG, if 6-[^18^F]FDOPA is not available) should be performed as first choices in VHL disease rather than SSTR PET or [^18^F]FDG for *SDHx*-related PPGL including metastatic disease (18, 19, 25) (**Figures 2**, **3**). [^18^F]FDG is sensitive in *SDHx*-PPGL, but images can be degraded by brown adipose tissue activation due to catecholamines oversecretion (25).

## Cushing syndrome

Cushing disease represents 75-80% of Cushing syndrome (CS) in older children; however, before 10 years of age, adrenocorticotrophic hormone (ACTH)-independent causes of CS are more common (27). McCune-Albright syndrome and primary pigmented nodular adrenocortical disease (PPNAD) are the two causes of ACTH-independent CS that are typically seen in children or young adults (27). The most common presentation of CS in children is growth retardation despite an increase in weight, except in patients with virilizing adrenal tumors, which may show growth acceleration (27–29). Hypertension and striae as well as other virilizing signs such as acne and hirsutism are seen in approximately 50% of patients (27–30). In children, headaches and fatigue are common. Whereas psychiatric and cognitive changes may affect school performance, these patients may show “compulsive diligence” and do quite well academically (27, 30, 31). McCune-Albright syndrome (post-zygotic mutations in the guanine nucleotide binding protein, alpha stimulating [*GNAS*] gene) can cause bilateral adrenocortical hyperplasia and represents the most common etiology of CS in neonates and young infants. Suspicion for the diagnosis can be raised by the presence of fibrous dysplasia of bone, café-au-lait spots, and the development of precocious puberty in girls (27, 32). This disorder is caused by an activating mutation of the α-subunit of the G protein–stimulating cyclic adenosine monophosphate (cAMP) formation at codon 201 (27).

The signs of endocrine excess manifesting as precocious puberty, CS, or virilization in the very young (<4 years) suggest adrenal carcinoma (ACC) (27, 33). ACC is a rare, aggressive, and malignant tumor arising from the adrenal cortex, and incidence of ACC is estimated to be three times more common than that of adenoma in childhood (ACC represents 5-6% of pediatric adrenal malignancies) (34, 35). These tumors have bimodal peaks (one at <5 years old and the other at 30-50 years old), and tumor size at the time of presentation is usually large (2.5–20 cm) (33). ACC should raise suspicion for Li-Fraumeni syndrome, Carney complex, Beckwith-Wiedemann syndrome, familial adenomatous polyposis coli, and MEN type 1 (33). Histopathologic examination cannot reliably differentiate between an adrenal adenoma and ACC, necessitating long-term follow-up even for histologically benign adrenal tumors (34, 36).

PPNAD, also known as micronodular adrenal disease, is characterized by small- or normal-sized adrenal glands with cortical micronodules (average 2–3 mm) that may be dark or black in color (27). PPNAD is often familial and associated with MEN syndrome and Carney complex. CS occurs only in 30% of cases of Carney complex and is caused by germline *PRKAR1A* mutations in up to 70% of cases (27). *PRKAR1A* (chromosome 17q22-24) encodes the protein kinase A (PKA) regulatory subunit type IA, an important regulator of cAMP signaling in most cells. PPNAD is usually diagnosed before the age of 30 years, with 50% of patients being younger than 15 years at diagnosis (37). In isolated PPNAD, germline mutations in *PRKAR1A* and in the phosphodiesterase 11A (*PDE11A*) gene have been demonstrated (38). In the pediatric population, ectopic ACTH secretion occurs rarely and is usually due to bronchial or thymic carcinoids (27, 39). Rare cases of adrenal oncocytomas have been reported.

Nuclear imaging studies are not needed for patients who have ACTH-dependent CS but are often required for those with the diagnosis of ACTH-independent CS. The studies are used mainly to distinguish unilateral from bilateral adrenal disease (40, 41) and to characterize large masses. Adrenal gland imaging is the mainstay in differentiating between the various types of ACTH-independent CS (27). In PPNAD, morphological imaging can show normal adrenal glands, bilateral micronodules, or eventual unilateral abnormality (micronodules with or without coexistence of macronodules) (42). Adrenal cortex functional imaging using NP-59 can be useful and typically shows a bilateral adrenal uptake, with possible asymmetrical uptake in patients with macronodules. The successful synthesis of PET-based NP-59 radiopharmaceutical, ^18^F-NP-59, and its superior imaging characteristics and reduced radiation dose compared to that of ^131^I-NP-59 will likely lead to NP-59’s adoption for adrenal cortex imaging in the future (43). In this situation, PET radiopharmaceuticals that target the CYP11B enzyme family are of interest and can reduce radiation dose exposure but have limited availability (44, 45).

**Figure 4:**
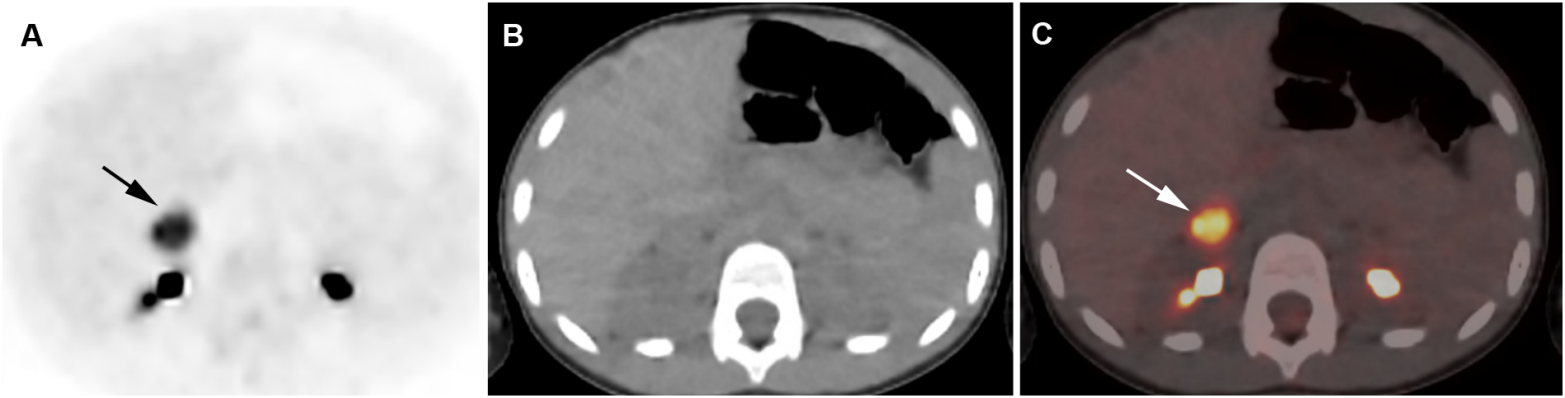
Adrenocortical adenoma. 5-year-old girl with history of neuroblastoma 4 years earlier. Suspected recurrence of neuroblastoma. Adrenal lesion noted on ultrasound as part of an evaluation of the urinary tract. [^18^F]FDG PET shows uptake in a right adrenal mass. Surgical resection of 2.5-cm adrenal adenoma. Transverse PET **(A)**, CT **(B)**, PET/CT fusion image **(C)**. Intense [^18^F]FDG uptake in a right adrenal mass (arrows).

**Figure 5:**
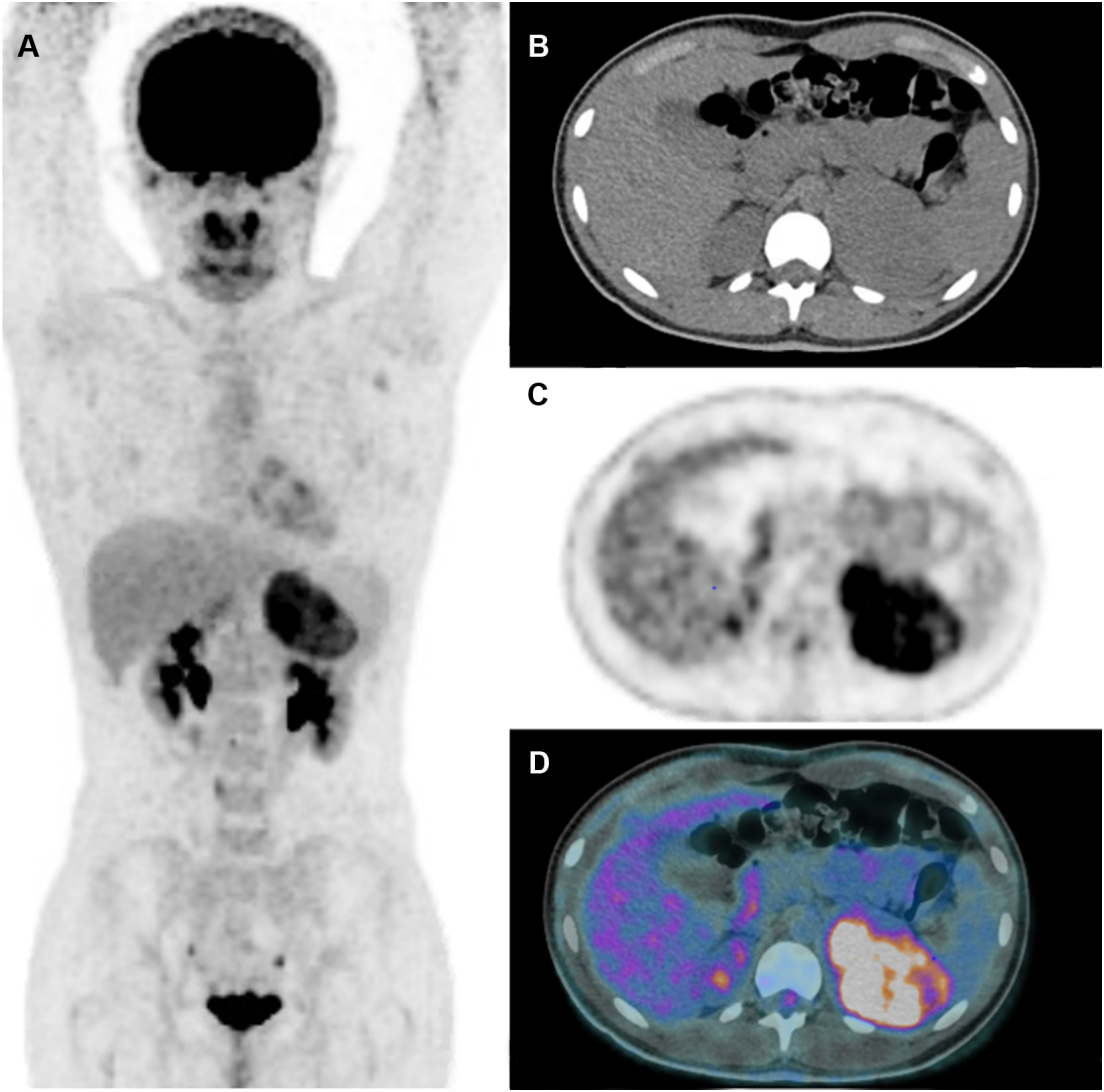
Adrenocortical carcinoma. 13-year-old girl with hyperandrogenism (secondary amenorrhea, hirsutism). Large adrenal mass on US and CT scan. [^18^F]FDG PET shows uptake in a left adrenal mass without metastasis. After surgery, pathological analysis confirmed the diagnosis of ACC with Wieneke score 3 (55), Ki-67 index 5.3% and p53-negative status. Complete remission 5 years after surgery. Maximum intensity projection image **(A)** Transverse CT **(B)**, PET **(C)**, PET/CT fusion image **(D)**. (Courtesy of Drs. Catherine Ansquer and Morgane Cleirec from Nantes University Hospital/Hôtel-Dieu).

The presence of a unilateral adrenal mass on CT/MRI should raise suspicion for ACC, especially if the lesion is >5 cm in diameter (27). ACC is typically a large heterogeneous tumor with necrosis and calcifications and can behave aggressively, with encasement of localized vascular structures and widespread metastasis. Signs of necrosis, hemorrhage, and calcification are characteristics of ACC and, less commonly, of PHEO, which can also co-secrete ACTH (46). In smaller tumors (<3 cm), MRI cannot accurately establish the benignity of adrenocortical tumors in pediatric patients. Criteria such as washout thresholds on CT scan and drop of signal on out-of-phase MRI cannot reliably predict the benignity of these tumors in children, as it does in the adult counterparts (34). [^18^F]FDG can be used for disease characterization and preoperative evaluation, and its revelations change the management plan in about 5% of ACC adult patients (47) (**Figures 4** and **5**). [^18^F]FDG can exhibit moderate to high uptake and has been shown useful for management of metastatic disease (48). A highly elevated tumor-to-liver uptake ratio can be observed in ACC with an oncocytic component or oncocytoma.

## Virilization or feminization

Adrenal virilism is due to an androgen-secreting adrenal tumor or adrenal hyperplasia (49). Virilization is more noticeable in girls (49). Clinical and auxological features cannot clearly distinguish adrenarche (e.g., onset of adrenal androgen secretion) from the other entities. Laboratory and imaging investigations are necessary to make a definite diagnosis (49). Adrenocortical tumors are rarely responsible for virilization and are most often characterized by rapid onset of clinical signs together with secretion of both glucocorticoids and androgens. Feminizing adrenal tumors (e.g., estrogen-secreting) are even more rare in childhood and can be caused by an oncocytoma that can be either benign or malignant. Although boys present with contrasexual pseudopuberty signs, girls present with isosexual pseudopuberty. Certain causes of virilization, such as true precocious puberty, testicular tumors, and congenital adrenal hyperplasia, can be excluded by history and physical examination (50). The major diagnostic problem often encountered is to preoperatively distinguish between adrenal and ovarian tumors in virilized females (50). Sustained elevation of ACTH in patients with congenital adrenal hyperplasia has been postulated to cause adrenal rest cells to grow and become functionally active. The so-called adrenal rest tissue may be seen at several sites throughout the body, including the celiac plexus region, broad ligaments, normal ovaries, and testes (51). In three pediatric series, more than half of the virilizing adrenal tumors were found to be carcinomas, indicating that such tumors may metastasize widely. Therefore, when an adrenal tumor is suspected, it is important to establish the correct diagnosis promptly (50).

Guided by the clinical findings and first laboratory results, imaging studies should be performed to exclude androgen-secreting adrenal tumor. Imaging techniques used for various clinical indications can detect incidental adrenal enlargement, and this may alert clinicians to the underlying subclinical conditions. The imaging modality of choice depends on the age of the child. Initial diagnosis of an adrenal mass can be made with ultrasound; however, it is operator dependent (52). Adrenal glands in patients with classical congenital adrenal hyperplasia are often enlarged (one limb >4 mm) and cerebriform (52, 53). The diagnosis of congenital adrenal hyperplasia is mainly based on clinical features and hormonal and genetic analysis. Morphological imaging has an important role in the diagnosis and management of these patients. It provides important information for the diagnosis, follow-up, compliance with treatment, and surgical planning. Ectopic adrenocortical masses should be considered in the differential diagnosis of other tumors, particularly when associated with hyperandrogenism in females. [^18^F]FDG PET imaging could help, too, for a precocious diagnosis of the adrenal tumor and its metastases, especially when other explorations fail to show the adrenal tumor (54).

## Adrenal incidentaloma

Adrenal incidentalomas are a rare finding in children. They can be revealed by calcification seen in the subdiaphragmatic regions or an adrenal mass on a US, CT, or MRI. Although common in adults, adrenal adenomas are very uncommon in children. Tumors are more likely to be derived from sympathetic ganglionic cells (neuroblastoma, ganglioneuroblastoma, and ganglioneuroma). Cysts, teratomas, or hematomas are very rare findings.

As for adults, hormone evaluations should be performed. Imaging should orient towards the diagnosis to avoid any unnecessary surgery. It is important to exclude potential pitfalls that could disguise an adrenal mass (e.g., prominent crus of diaphragm or lipoma of diaphragmatic crus, fat in suprarenal fossa, subdiaphragmatic extralobular pulmonary sequestration, and extramedullary hematopoiesis that may occur in children with ineffective RBC production) (34).

Sympathetic tumors cannot be distinguished from each other *via* imaging alone, and the diagnosis must be established based on histopathological analysis. Nuclear medicine studies using specific radiopharmaceuticals are most useful in endocrine active lesions or, in select cases, for tumor characterization and to exclude metastases.

## Conclusion

The adrenal glands in pediatric patients can be affected by a variety of neoplasms. Imaging plays a crucial role in identifying and differentiating malignant and benign adrenal neoplasms. The diagnosis of adrenal lesions can be challenging; however, knowledge about clinical presentations and the multimodality imaging characteristics of different adrenal neoplasms can lead to an accurate diagnosis and may direct biopsy or surgery. Multimodality imaging helps to define the origin, extent, and relationship of these lesions to adjacent structures and to guide treatment management.

## Author contributions

All authors contributed to the design and implementation of the research, to the analysis of the literature and to the writing of the manuscript. All authors contributed to the article and approved the submitted version.
